# The Contribution of Genetic Variants to the Risk of Papillary Thyroid Carcinoma in the Kazakh Population: Study of Common Single Nucleotide Polymorphisms and Their Clinicopathological Correlations

**DOI:** 10.3389/fendo.2020.543500

**Published:** 2021-01-22

**Authors:** Zhanna Mussazhanova, Tatiana I. Rogounovitch, Vladimir A. Saenko, Ainur Krykpayeva, Maira Espenbetova, Bauyrzhan Azizov, Hisayoshi Kondo, Katsuya Matsuda, Zhanna Kalmatayeva, Raushan Issayeva, Zhanar Yeleubayeva, Madina Madiyeva, Aray Mukanova, Marat Sandybayev, Saltanat Bolsynbekova, Zhanna Kozykenova, Shunichi Yamashita, Masahiro Nakashima

**Affiliations:** ^1^ Department of Tumor and Diagnostic Pathology, Atomic Bomb Disease Institute, Nagasaki University, Nagasaki, Japan; ^2^ Faculty of Medicine and Health Care, Al-Farabi Kazakh National University, Almaty, Kazakhstan; ^3^ Department of Radiation Medical Sciences, Atomic Bomb Disease Institute, Nagasaki University, Nagasaki, Japan; ^4^ Department of Radiation Molecular Epidemiology, Atomic Bomb Disease Institute, Nagasaki University, Nagasaki, Japan; ^5^ Department of Endocrinology, Semey Medical University, Semey, Kazakhstan; ^6^ Endovascular Laboratory of Training Hospital, Semey Medical University, Semey, Kazakhstan; ^7^ Biostatics Section, Division of Scientific Data Registry, Atomic Bomb Disease Institute, Nagasaki University, Nagasaki, Japan; ^8^ Center of Morphological Examination, Kazakh Institute of Oncology and Radiology, Almaty, Kazakhstan; ^9^ Radiology and Nuclear Medicine, Semey Medical University, Semey, Kazakhstan; ^10^ Center of Nuclear Medicine and Oncology of Semey, Semey, Kazakhstan; ^11^ Department of Pathological Physiology, Semey Medical University, Semey, Kazakhstan

**Keywords:** papillary thyroid carcinoma, single nucleotide polymorphism, case–control genetic association study, risk factors for thyroid cancer, clinicopathological correlations

## Abstract

**Objective:**

Risk for developing papillary thyroid carcinoma (PTC), the most common endocrine malignancy, is thought to be mediated by lifestyle, environmental exposures and genetic factors. Recent progress in the genome-wide association studies of thyroid cancer leads to the identification of several genetic variants conferring risk to this malignancy across different ethnicities. We set out to elucidate the impact of selected single nucleotide polymorphisms (SNPs) on PTC risk and to evaluate clinicopathological correlations of these genetic variants in the Kazakh population for the first time.

**Methods:**

Eight SNPs were genotyped in 485 patients with PTC and 1,008 healthy control Kazakh subjects. The association analysis and multivariable modeling of PTC risk by the genetic factors, supplemented with rigorous statistical validation, were performed.

**Result:**

Five of the eight SNPs: rs965513 (*FOXE1*/*PTCSC2*, *P* = 1.3E-16), rs1867277 (*FOXE1* 5’UTR, *P* = 7.5E-06), rs2439302 (*NRG1* intron 1, *P* = 4.0E-05), rs944289 (*PTCSC3*/*NKX2-1*, *P* = 4.5E-06) and rs10136427 (*BATF* upstream*, P* = 9.8E-03) were significantly associated with PTC. rs966423 (*DIRC3*, *P* = 0.07) showed a suggestive association. rs7267944 (*DHX35*) was associated with PTC risk in males (*P* = 0.02), rs1867277 (*FOXE1*) conferred the higher risk in subjects older than 55 years (*P* = 7.0E-05), and rs6983267 (*POU5F1B/CCAT2*) was associated with pT3–T4 tumors (*P* = 0.01). The contribution of genetic component (unidirectional independent effects of rs965513, rs944289, rs2439302 and rs10136427 adjusted for age and sex) to PTC risk in the analyzed series was estimated to be 30–40%.

**Conclusion:**

Genetic factors analyzed in the present work display significant association signals with PTC either on the whole group analysis or in particular clinicopathological groups and account for about one-third of the risk for PTC in the Kazakh population.

## Introduction

Papillary thyroid carcinoma (PTC), a well-differentiated thyroid cancer of follicular cell origin, accounts for about 80% of all thyroid cancers worldwide being the most common endocrine malignancy. According to the IARC, in 2018 thyroid cancer affected 567,233 patients worldwide, making it the 9^th^ most prevalent human cancer (3.1%) with the average age-standardized incidence of 6.7 and mortality rate of 0.42 per 100,000 of population (1). Region-specific incidence rates vary broadly from 1.0 in Micronesia to 15.0 in North America per 100,000 of population. In Kazakhstan, the age-standardized incidence of thyroid cancer was 2.4 per 100,000 of population accounting for 1.4% of all newly diagnosed cancers in the country in 2018.

With the improvements in cancer detection and diagnosis, the incidence of thyroid cancer is growing in most countries displaying one of the fastest increases in rate among common cancers. While the advances in thyroid nodule visualization such as ultrasound imaging and their facile assessment using ultrasound-guided fine-needle aspiration cytology have likely contributed to this uptrend, the additional reasons for the increasing incidence are investigated. Besides of well-established risk factor for thyroid cancer such as ionizing radiation, other environmental agents, including iodine deficiency, natural and technogenic pollutants with hormone disrupter effects, exposures to excessive nitrate (2, 3) and various chemicals are discussed or considered.

As a complex disease, PTC is thought to be dependent not only on environmental, but also on genetic factors. Studies of familial thyroid cancer estimated the contribution of genetic component to the risk of disease to be ranging from 28 to 53% (4, 5). At the population level, hereditary factors possibly contributing to the phenotype (*e.g.* the development of a condition or a disease) are usually identified in genetic association studies. To date, a number of well-powered genome-wide association studies (GWAS) or target gene investigations in thyroid cancer have been performed in the groups of different ethnicities in non-exposed or exposed to radiation individuals (6–14). GWAS findings and consequent independent replication studies have convincingly demonstrated robust associations of rs965513 (*FOXE1*, forkhead box E1 and/or *PTCSC2*, papillary thyroid carcinoma susceptibility candidate 2; chromosome 9q22.33), rs944289 (*PTCSC3*, papillary thyroid carcinoma susceptibility candidate 3 and/or *NKX2-1*, NK2 homeobox 1; 14q13.3), rs1867277 (*FOXE1;* 9q22.33), rs2439302 (*NRG1*, neuregulin 1; 8q12) and rs966423 (*DIRC3*, disrupted in renal carcinoma 3; 2q35) SNPs with differentiated thyroid cancer, principally PTC (15–27), reviewed in (28). The strength of association signal for these SNPs in terms of odds ratios (OR) ranged from 1.28 to 1.70 in most studies. More recent studies have identified associations between the rs6983267 (*POU5F1B*, POU class 5 homeobox 1B and/or *CCAT2*, colon cancer associated transcript 2; 8q24) and thyroid cancer in different populations. A systematic review with meta-analysis of four studies that included a total of 2,825 cases and 9,684 controls confirmed the G allele of the rs6983267 to be a risk factor for thyroid cancer with an OR = 1.08, *P* = 0.01 (29).

The novel GWAS candidate loci continue to emerge. A recent combined analysis of GWAS results and the Italian replication study provided evidence of association of risk for differentiated thyroid cancer with rs10136427 (*BATF*, basic leucine zipper ATF-like transcription factor, 14q24.3) with an OR = 1.40, *P* = 4.35E-07) and rs7267944 (*DHX35*, DEAH-box helicase 35, 20q12) with an OR 1.39, *P* = 2.13E-08. These associations were replicated in the Polish and Spanish populations with little evidence of population heterogeneity (the combined, OR = 1.30, *P* = 9.30E-07 and OR 1.32, *P*=1.34E-08, respectively) (10).

To the best of the authors’ knowledge, studies of rs10136427 (*BATF*, 14q24.3) and rs7267944 (*DHX35*, 20q12) in PTC have not been replicated in independent studies. We therefore aimed to examine the six well-described SNPs discussed above, namely rs965513 (*FOXE1*/*PTCSC2*, 9q22.33), rs944289 (*PTCSC3*/*NKX2-1*, 14q13.3), rs1867277 (*FOXE1;* 9q22.33), rs2439302 (*NRG1*, 8q12), rs966423 (*DIRC3*, 2q35) and rs6983267 (*POU5F1B/CCAT2*, 8q24.2), and two SNPs newly discovered to be associated with thyroid cancer (10), rs10136427 (*BATF*, 14q24.3) and rs7267944 (*DHX35*, 20q12) in a relatively large case–control series. This work is the first to characterize the eight SNPs in the Kazakh population. In addition to the classical association analysis, we estimated the contribution of the genetic variants to PTC risk, and assessed the relationships with clinicopathological characteristics of tumors in the study since available information is very limited in the literature.

## Material and Methods

### Study Population

A total of 485 patients with histologically confirmed PTC (90.3% females, mean age 54.78 ± 13.3 y.o., 18–87 y.o., range) operated from 1980 to 2015, and 1,008 healthy control subjects (78.7% females, mean age 39.0 ± 15.8 y.o., 15–83 y.o., range) of Kazakh origin were recruited. Clinicopathological information was retrieved from medical records (**Table 1**). The pathological classification was based on the World Health Organization definitions (30), pathological staging (pTNM, where the T category defined the anatomic extent of cancer for the tumor, N for the lymph nodes and M for distant metastases) was according to the 7th edition of TNM classification system (31). Patients and control subjects had no history of radiation exposure. All participants or their parents/guardian gave written informed consent in accordance with the Declaration of Helsinki. A peripheral venous blood sample was collected from each participant. The protocol of this study was approved by corresponding ethics committees.

**Table 1:** Demographic characteristics of control subjects and PTC patients, and clinicopathological data.

Characteristics	Value (%)^1^
*Healthy control subjects, n = 1,008*	
**Age at sampling**, M ± SD (range)	39.0 ± 15.8 (15–83)
**Sex**	
Female	793 (78.7)
Male	215 (21.3)
*PTC patients, n = 485*	
**Age at diagnosis**, M ± SD (range)	54.8 ± 13.3 (18–87)
**Sex**	
Female	438 (90.3)
Male	47 (9.7)
**pT** ^2^	
T1	98 (20.2)
T2	264 (54.4)
T3	85 (17.5)
T4	38 (7.8)
**N category** ^2^	
N0	259 (53.4)
N1	74 (15.3)
NX	152 (31.3)
**M category** ^2^	
M0	376 (77.5)
M1	2 (0.4)
MX	107 (22.1)

^1^Mean ± standard deviation and (range) for age in years, count data and (%) for other variables.

^2^The pathological cancer staging (pTNM, where the T category defines the anatomic extent of cancer for the tumor, N for the lymph nodes and M for distant metastases; 0,1 and X in the N and M categories correspond to absent, present, and unknown, respectively) according to the 7th edition of TNM classification system (31).

### DNA Isolation and Genotyping

Blood DNA was extracted using QIAamp DNA Mini Kit (QIAGEN, Tokyo, Japan) according to the manufacturer’s protocol. DNAs of sufficient quality for genotyping were obtained from all 485 PTC patients and 1,008 control participants.

Genotyping was performed with predesigned Custom Applied Biosystems TaqMan SNP Genotyping Assays (**Table 2**) using TaqMan Genotyping Master mix (all reagents from ThermoFisher Scientific) and 10 ng genomic DNA per 10 µl reaction in a Light Cycler 480 (Roche, Indianapolis, IN). Cycling conditions were as follows: denaturation at 95°C for 10 min followed by 60 cycles of 92°C for 15 s and 62°C for 1 min for all SNPs. As a quality control, 15–20% of all samples were randomly selected and re-run in duplicates for each SNP. Full concordance between the experiments was observed.

**Table 2:** TaqMan primer/probe set used for genotyping.

SNP	Chromosomal locus	Base position^1^	Nearest gene(s)	TaqMan primer/probe set
rs965513 (A/G)	9q22.33	97,793,827	*FOXE1*, *PTCSC2*	C_1593670_20
rs944289 (T/C)	14q13.3	36,180,040	*NKX2-1*, *PTCSC3*	C_1444137_10
rs1867277 (A/G)	9q22.33	97,853,632	*FOXE1*	C_11736668_10
rs2439302 (G/C)	8q12	32,574,851	*NRG1*	C_16238367_10
rs10136427 (C/T)	14q24.3	75513546	*BATF*	C_2676717_10
rs966423 (T,G/C)	2q35	217,445,617	*DIRC3*	C_1880230_10
rs7267944 (C/T)	20q12	39,318,791	*DHX35*	C_29372376_10
rs6983267 (G/T)	8q24.2	127,401,060	*POU5F1B, CCAT2*	C_29086771_20

^1^Genomic location of an SNP position on GRCh38.

### Statistical Analyses

#### Association Analysis

We used PLINK 1.9 (32) software to run the multiplicative genetic models in the case–control sample for each SNP with age and sex as covariates. This type of model evaluates the impact of individual alleles of a polymorphic locus on the disease. The multiplicative models have been used in the vast majority of the genome-wide and replication association studies of thyroid cancer (6–27); using those in our work provided an opportunity to compare the strength of association signals (ORs) between the previous studies and our findings. The risk alleles were assigned according to the cited literature sources for consistency; summary information on the risk alleles is provided in **Table 3**. Multiple testing correction (the Benjamini–Hochberg method) and the adaptive label-swapping permutation test (10^6^, maximum) were performed using options available in PLINK.

**Table 3:** Summary information on the risk alleles of analyzed SNPs.

SNP	Chromosomal locus	Nearest gene(s)	Location/annotation	Risk allele function^1^	Risk allele frequency^1^	Allelic OR^1^	Replication studies	References
rs965513 (A*/G)	9q22.33	*FOXE1, PTCSC2*	Intergenic, long-range enhancer	Decreases the expression of *FOXE1*, unspliced *PTCSC2* and *TSHR* in normal thyroid tissue	0.09–0.61	1.40–2.81	Yes	(6–9, 16–18, 20–27, 33, 34)
rs944289 (T*/C)	14q13.3	*PTCSC3, NKX2-1*	Non-coding, promoter	Decreases *PTCSC3* expression by destroying a C/EBP*α*/*β* transcription factor binding site in *PTCSC3* promoter	0.46–0.70	1.12–1.60	Yes	(6, 8, 18, 20, 21, 23–27, 35)
rs1867277 (A*/G)	9q22.33	*FOXE1*	5’UTR	Upregulates *FOXE1* expression in follicular thyroid carcinoma cells through the recruitment of USF1/USF2 transcription factors	0.16–0.53	1.20–1.75	Yes	(15, 18, 20, 23, 25, 26)
rs2439302 (G*/C)	8p12	*NRG1*	Intron 1	Increases *NRG1* expression	0.23–0.54	1.29–1.53	Yes	(8, 24, 27, 33, 36)
rs10136427 (C*/T)	14q24.3	*BATF*	Intergenic	Not established	0.79–0.88	1.05–1.62	No	(10)
rs966423 (T,G*/C)	2q35	*DIRC3*	Intron	Not established	0.41–0.82	1.14–1.28	Yes	(8, 24, 27, 33)
rs7267944 (C*/T)	20q12	*DHX35*	Intergenic	Not established	0.17–0.26	1.10–1.54	No	(10)
rs6983267 (G*/T)	8q24.2	*POU5F1B, CCAT2*	Non- coding	Suggested to predispose chromosome 8 to chromosomal instability	0.37–0.51	0.89–1.16	Yes	(23, 24, 26, 29)

^1^Information from the literature sources.

*The risk allele is indicated by an asterisk.

Associations between each SNP and clinicopathological parameters of PTCs were assessed using logistic regression models with binary outcomes sex (F *vs* M), age (≥55 *vs <*55 years old), pathological tumor (pT) category (pT3 or pT4 *vs* pT1 or pT2) or nodal disease (N1 *vs* N0, *i.e.* present *vs* absent) as dependent variables, and individual SNPs, age and sex (where applicable) as explanatory variables. The LOGISTIC procedure in the 3.71 release of SAS Studio for the 9.4M5 version of SAS (SAS Institute, Cary, NC, USA) was used for these calculations.

Exact two-sided tests, permutation tests and exact test for equality of allele frequencies for stratified groups were performed using the ‘HardyWeinberg’ package in R (37).

All tests were two-sided, *P <*0.05 was considered statistically significant.

### Predictive Modeling of Papillary Thyroid Carcinoma

To evaluate the impact of the genetic component on PTC risk in the given case–control sample, we used multivariable logistic regression modeling. The initial full model included all eight SNPs in the study, and age and sex as explanatory variables. The reduced model was determined by stepwise or non-automatic selection of variables to achieve minimum Akaike information criterion. Statistical validation of the reduced model was performed using permutation analysis as described before (38), and bootstrapping with 0.9 sampling rate (*i.e.*, selecting 90% of data for each sample using the unrestricted random sampling method) in 10,000 replicates using the SURVEYSELECT procedure. The receiver operating characteristic (ROC) analysis was performed to assess the predictive performance of the reduced model, supplemented with the leave-one-out cross-validation.

## Results

### Single_Nucleotide Polymorphisms Association With and Impact on Papillary Thyroid Carcinoma Risk

Five of the eight SNPs displayed significant association signals in the Kazakh population with ORs similar to those in the original studies and follow-up publications (**Table 4**). The strongest associations between a risk allele and sporadic PTC were observed for rs965513 (*FOXE1*/*PTCSC2*, 9q22.33; OR = 2.25, *P* = 1.3E-16), rs1867277 (*FOXE1* 5’UTR, 9q22.33; OR = 1.52, *P* = 7.5E-06), rs2439302 (*NRG1* intron 1, 8q12; OR = 1.46, *P* = 4.0E-05), rs944289 (*PTCSC3*/*NKX2-1*, 14q13.3; OR = 1.44, *P* = 4.5E-06), and rs10136427 (intergenic region upstream *BATF*, 14q24.3; OR = 1.30, *P* = 9.8E-03). rs966423 (*DIRC3*, 2q35) showed a significant association (OR = 1.25, *P* = 5.8E-03) on unadjusted analysis, but significance became marginal after adjusting for age and sex (OR = 1.18, *P* = 0.07). Adjustment for multiple testing and statistical validation (permutation) confirmed significant association of the five SNPs (rs965513, rs944289, rs1867277, rs2439302, rs10136427) and suggestive association for rs966423.

**Table 4:** Association analysis of papillary thyroid carcinoma in the Kazakh population.^1^

SNP^2^	Locus	Nearest gene(s)	Risk allele frequency		OR [95%CI]	*P*-value	FDR^3^	*P*-permutation
			Cases n = 485	Controls n = 1008				
rs965513[A]	9q22.33	*FOXE1*, *PTCSC2*	0.39	0.22	2.25 [1.86–2.73]	1.3E-16	1.1E-15	1.0E-06
rs944289[T]	14q13.3	*NKX2-1*, *PTCSC3*	0.55	0.46	1.44 [1.21–1.72]	4.6E-05	9.1E-05	3.4E-05
rs1867277[A]	9q22.33	*FOXE1*	0.43	0.32	1.52 [1.27–1.83]	7.5E-06	3.0E-05	7.0E-06
rs2439302[G]	8q12	*NRG1*	0.43	0.33	1.46 [1.22–1.76]	4.0E-05	9.1E-05	4.6E-05
rs10136427[C]	14q24.3	*BATF*	0.77	0.72	1.30 [1.07–1.59]	9.8E-03	0.02	0.01
rs966423[T,G]	2q35	*DIRC3*	0.40	0.35	1.18 [0.98–1.42]	0.07	0.10	0.09
rs7267944[C]	20q12	*DHX35*	0.23	0.24	1.04 [0.85–1.28]	0.71	0.71	0.70
rs6983267[G]	8q24.2	*POU5F1B, CCAT2*	0.48	0.48	1.09 [0.91–1.30]	0.36	0.41	0.44

^1^The multiplicative model adjusted for age and sex.

^2^The risk allele is specified in brackets.

^3^False discovery rate, the Benjamini–Hochberg procedure.

Two remaining SNPs, rs7267944 (*DHX35*, 20q12; OR = 1.04, *P* = 0.71) and rs6983267 (*POU5F1B/CCAT2*, 8q24.2; OR = 1.09, *P* = 0.36) did not display significant associations at this stage of analysis.

After obtaining evidence that certain examined SNPs display statistically significant association signals, we set out to determine the performance of a statistical model of the risk for PTC based on genetic factors. The reduced model included four SNPs: rs965513 (*FOXE1*/*PTCSC2*), rs944289 (*NKX2-1*, *PTCSC3*), rs2439302 (*NRG1*) and rs10136427 (*BATF*) (**Table 5**). Their association signals remained significant after correction for multiple testing (Bonferroni and FDR). Statistical validation confirmed significant association of these SNPs with cancer risk (permutation), and confidence intervals almost did not change on bootstrapping. Of note, the OR estimates for the four SNPs in the model were very similar to those obtained in the single-SNP models (see **Table 4**) indicative of independent contribution of each SNP to thyroid cancer risk. The area under the ROC curve (AUC) was 0.82 (95%CI 0.80–0.84; *P* = 3.2E-183 as compared with a random classifier); cross-validation did not demonstrate model overfit returning a similar AUC of 0.82 (0.80–0.84). The Cox and Snell pseudo-R^2^ of the optimal model was 0.27, and the Nagelkerke pseudo-R^2^ was 0.38 suggesting that some 30–40% of variability in the risk for PTC in the analyzed series could be explained by the model that included age, sex and the four SNPs.

**Table 5:** The SNP-based logistic regression model of risk for PTC in the Kazakh population.^1^

SNP	Logistic regression		Multiple testing		Permutation	Bootstrap^2^
	OR [95%CI]	*P*-value	Bonferroni	FDR	*P*-value	OR [95%CI]
rs965513	2.32 [1.91–2.83]	4.8E-17	2.4E-16	1.1E-16	<1.0E-04	2.36 [1.98–2.81]
rs944289	1.43 [1.19–1.72]	1.4E-04	2.8E-04	1.6E-04	1.0E-04	1.44 [1.22–1.68]
rs2439302	1.50 [1.24–1.82]	2.9E-05	8.8E-05	4.1E-05	<1.0E-04	1.51 [1.28–1.79]
rs10136427	1.32 [1.06–1.63]	1.1E-02	1.1E-02	1.1E-02	6.8E-03	1.33 [1.09–1.61]

^1^The multiplicative model adjusted for age and sex.

^2^Bootstrap sampling rate 0.9, 10,000 replicates.

### Single-Nucleotide Polymorphism Association With Clinicopathological Parameters of Papillary Thyroid Carcinoma

We assessed the genetic variants association with patients’ sex, age (older than 55 years old), the pT category (pT3–T4 *vs* pT1–T2), and regional metastasis (N1). As shown in **Table 6**, very few statistically significant associations were found. rs7267944 (*DHX35*) was negatively associated with female sex (OR = 0.40, *P* = 6.1E-05), rs1867277 (*FOXE1*) was weakly but nominally significantly associated with the older patients’ age (OR = 1.32, *P* = 0.03) and rs6983267 (*POU5F1B/CCAT2*) with more advanced tumors (OR = 1.49, *P* = 0.01).

**Table 6:** Association of the genetic variants with clinicopathological characteristics.^1^

SNP	Sex (F)			Age ≥ 55 y.o.			pT3–T4 *vs* pT1–T2^2^			N1 *vs* N0^3^		
	*P*-trend^4^	OR^5^[95% CI]	*P*-value	*P*-trend	OR^6^[95% CI]	*P*-value	*P*-trend	OR[95% CI]	*P*-value	*P*-trend	OR[95% CI]	*P*-value
rs965513[A]^7^	0.76	1.07 [0.70–1.63]	0.76	0.09	1.25 [0.97–1.61]	0.08	0.87	1.02 [0.77–1.36]	0.88	0.50	1.14 [0.80–1.63]	0.47
rs944289[T]	0.27	1.00 [0.97–1.02]	0.71	0.25	1.18 [0.91–1.52]	0.22	0.10	0.78 [0.58–1.04]	0.09	0.78	0.96 [0.66–1.41]	0.83
rs1867277[A]	0.55	1.15 [0.75–1.75]	0.53	**0.04**	**1.32** **[1.02**–**1.69]**	**0.03**	0.33	0.85 [0.64–1.13]	0.27	1.00	1.02 [0.71–1.47]	0.90
rs2439302[G]	0.06	1.52 [1.00–1.02]	0.06	0.70	0.97 [0.76–1.24]	0.79	0.37	0.88 [0.66–1.16]	0.35	0.13	0.78 [0.54–1.11]	0.17
rs10136427[C]	0.45	0.82 [0.48–1.40]	0.47	0.28	1.17 [0.87–1.59]	0.17	0.82	1.02 [0.72–1.45]	0.90	0.98	1.00 [0.64–1.57]	0.99
rs966423[T,G]	0.58	1.15 [0.72–1.82]	0.56	0.46	1.11 [0.85–1.45]	0.43	0.89	0.96 [0.70–1.30]	0.80	0.90	1.04 [0.70–1.54]	0.84
rs7267944[C]	**3.4E-05**	**0.40** **[0.26**–**0.63]**	**6.1E-05**	0.41	0.84 [0.32–1.13]	0.11	0.79	0.95 [0.67–1.35]	0.79	0.96	0.96 [0.60–1.51]	0.84
rs6983267[G]	0.08	1.00 [0.98–1.02]	0.80	0.68	0.96 [0.74–1.25]	0.76	**0.02**	**1.49** **[1.09**–**2.02]**	**0.01**	0.66	1.10 [0.74–1.64]	0.63

^1^Multivariate logistic regression adjusted for age and sex unless otherwise specified.

^2^The T category (defines the anatomic extent of cancer for the tumor) from the pathological cancer staging according to the 7th edition of TNM classification system (31); here, the advanced tumors (pT3–T4) are contrasted to less advanced tumors (pT1–T2).

^3^The N category (defines the regional lymph node involvement) from the pathological cancer staging according to the 7th edition of TNM classification system (31); here, cases with nodal disease (N1) are contrasted to those without lymph node involvement (N0).

^4^The Cochran–Armitage test for trend.

^5^Adjusted for age.

^6^Adjusted for sex.

^7^The risk allele is specified in brackets. Statistically significant associations are shown in bold.

The strong association of rs7267944 (*DHX35*) with patients’ sex prompted us to test its association with PTC risk using stratified sampling. While no association was found in females OR = 0.95 (95%CI 0.76–1.18, *P* = 0.62 adjusted for age), the association signal in males was significant with an OR = 1.83 (95%CI 1.09–3.09, *P* = 0.02 adjusted for age). The difference in effect size was statistically significant (*P* = 0.023, the Breslow–Day test). No deviations from Hardy–Weinberg equilibrium in the groups of PTC patients or healthy control subjects, either non-stratified or stratified by sex, was found for this genetic variant (*P* > 0.4 for any exact two-sided test, *P* > 0.4 for any permutation test). Exact test for equality of allele frequencies for males and females in the control subjects was negative (*P* = 0.06), but in PTC patients a strong inequality was observed (*P* = 8.26E-05), in line with other statistical findings.

The modifying effect of age on rs1867277 (*FOXE1*) was tested in respective groups of patients and control subjects younger or older 55 years old. In the younger group, rs1867277 displayed an association signal with OR = 1.44 (95%CI 1.16–1.80, *P* = 9.5E-04 adjusted for sex), and in the older group with OR = 1.84 (95%CI 1.36–2.49, *P* = 7.0E-05 adjusted for sex). There was no surprise that the association was significant in both age groups as rs1867277 was significant on the whole group association analysis, which was adjusted for age (see **Table 4**). Clearly, the effect of rs1867277 on the risk for PTC was more pronounced in subject older than 55 years old, although the difference did not reach statistical significance (*P* = 0.20). No deviations from Hardy–Weinberg equilibrium in the groups of PTC patients or healthy control subjects were found for rs1867277 (*P* > 0.1 for any exact two-sided test, *P* > 0.1 for any permutation test). Exact test for equality of allele frequencies for subjects younger than 55 years old was negative (*P* = 0.85), while in older subjects the inequality existed (*P* = 0.03).

We also tested the association of rs6983267 (*POU5F1B/CCAT2*) with PTC of different pT stage. In pT1–T2 tumors, the association was insignificant with OR = 1.0 (95%CI 0.82–1.21, *P* = 0.96 adjusted for age and sex), while in pT3–T4 tumors the signal was significant, OR = 1.47 (95%CI 1.09–1.98, *P* = 0.01 adjusted for age and sex). The difference in ORs was statistically significant (*P* = 0.03). No deviations from Hardy–Weinberg equilibrium in the groups of PTC patients or healthy control subjects were found for rs6983267 (*P* > 0.1 for any exact two-sided test, *P* > 0.1 for any permutation test). Exact test for equality of allele frequencies in the subgroup of PTCs of pT1–pT2 category was negative (*P* = 0.32) and marginally significant in pT3–pT4 PTCs (*P* = 0.07).

## Discussion

In the present study we set out to determine the impact of genetic factors on PTC risk in the Kazakh population. We focused on several SNPs found to display confident association signals in the previous studies across different populations/ethnicities and also tested two SNPs that have been newly discovered in GWAS of thyroid cancer.

Our genotyping results unambiguously confirmed the associations of rs965513 (*FOXE1*/*PTCSC2*, 9q22.33), rs1867277 (*FOXE1* 5’UTR, 9q22.33), rs944289 (*PTCSC3*/*NKX2-1*, 14q13.3), rs2439302 (*NRG1* intron 1, 8q12) using canonical multivariate analysis essentially supplemented by rigorous statistical validation.

Functional roles of rs965513 and rs1867277 located in the *FOXE1* locus on chromosome 9q22.33 were linked to the transcriptional regulation of *FOXE1* and *PTCSC2*. rs965513 was shown to affect the expression of *FOXE1*, *PTCSC2* and *TSHR* (thyroid stimulating hormone receptor) in thyroid tissue (34), and rs1867277 regulates *FOXE1* expression through the recruitment of USF1/USF2 transcription factors (15), implicating these SNPs into thyroid homeostasis and development. Of note, transgenic mice overexpressing FOXE1 in their thyroids displayed retardation in the proliferation of follicular cells, suggestive of its tumor suppressor function (39). A meta-analysis that combined data from 23 studies in different countries and ethnicities evaluated that rs965513[A] risk allele had an OR = 1.58 (95% CI 1.32–1.90) in the pooled sample, and OR = 1.65 and 1.49 in Caucasian and Asian populations, respectively (40). Interestingly, in the Kazakh population, which is of Asian descent, we found an OR = 2.25 (95% CI 1.85–2.73), which is one of the strongest association signals ever reported for the *FOXE1* locus. Also of interest is the finding of age-related effect rs1867277 (*i.e.*, the higher risk in patients aged more than 55 years), which is reported for the first time. It should be noted that despite rs965513 and rs1867277 are located in the same genetic locus, their effect on PTC risk is independent (41). The age relatedness of rs1867277 effect could be addressed in independent or already available studies.

rs944289 located on chromosome 14q13.3 regulates expression of the *PTCSC3* lncRNA, which has tumor suppressor effect in thyroid cancer cell lines, through the recruitments of C/EBP*α* and *β* transcription factors (35). *PTCSC3* level was found to be significantly lower in PTC than in normal thyroid tissue (26), which corresponds well with its tumor suppressor function. Interestingly, rs944289 besides of PTC was also associated with follicular adenoma (26), indicating its broader function in thyroid tumorigenesis.

rs2439302 is located in intron 1 of *NRG1* on chromosome 8q12. *NGR1* encodes human epidermal growth factor receptor 3 (HER3) ligand whose dimers with HER2 can activate MAPK and AKT pathways known to play an important role in PTC (42). Similarly to *PTCSC3*, NRG1 was also earlier associated with follicular adenoma (26).

rs10136427, whose association with PTC risk was confirmed in the Kazakh population for the first time, was previously detected on GWAS in the Italian, Polish, and Spanish population study providing strong evidence of association with DTC (12). rs10136427 is located in an intergenic region upstream *BATF*. BATF proteins are the “AP-1 inhibitors”; findings in mouse myeloid leukemia cells suggested they can act as tumor suppressors by promoting cell growth arrest and cell differentiation. Whether BATF could play a similar role in other tissues, such as the thyroid, remains unknown. Since this genetic variant was not immediately associated with *BATF* expression, a possibility was suggested that this genetic locus may act as a *trans*-regulatory region controlling the expression of distant genes that reside on the same or even different chromosome(s) (*trans*-eQTL) (12).

rs966423 located in the *DIRC3* intron on chromosome 2q35, displayed marginally significant association (*P* = 0.07) in the Kazakh population. This genetic variant was significantly associated with the risk for thyroid cancer in both European and Asian ethnicities with ORs from 1.28 to 1.34 (reviewed in (28). In our study the OR = 1.18 (adjusted for age and sex) is lower than those previously described. It therefore is likely that our sample size (485 PTC patients and 1,008 healthy control subjects) did not provide sufficient statistical power (achieved power 44%). We interpret the association signal of this genetic variant as suggestive in the Kazakh population. The functional role of *DIRC3* lncRNA is likely to be that of tumor suppressor, and its relevance to thyroid cancer (8, 9) and other human malignancies such as, originally, familial renal cancer (43), melanoma (44), breast cancer (45) and laryngeal squamous cell carcinoma (46) has been reported.

The finding for rs7267944, which is located approximately 280 kB telomeric to *DHX35* on chromosome 20q12, was somewhat unexpected. While on the whole group association analysis the signal of rs7267944 was insignificant, we noticed a strong modifying effect of sex. Accordingly, we found significant association of rs7267944 with PTC in males but not in females. *DHX35* encodes a putative RNA helicase of DEAD/DEAH-box family, which are implicated in translation initiation, RNA splicing, and ribosome and spliceosome assembly. *DHX35* is relatively highly expressed in the endometrium, ovaries, prostate and testes possibly pointing at its relatedness to sex-specific biological function (47). In the thyroid, *DHX35* is also expressed, although its role in tissue homeostasis and carcinogenesis remains unestablished. Within our study we could not determine the reason for *DHX35* association with sex, which, besides the true association could be sampling bias, a phenomenon specific for the given ethnic group (and relevant environmental exposures) or occurring by chance. This could be clarified in an independent study in the Kazakh population and also in other ethnic groups by researcher with access to rs7267944 [or other SNP(s) in strong or perfect linkage disequilibrium with it] genotyping data and clinical/demographic information. It is also possible that rs7267944 may point on the genetic factor other than *DHX35* on chromosome 20q12 or elsewhere due to *trans*-eQTL effect.

A recent GWAS has identified rs6983267 (*POU5F1B/CCAT2*) as a key locus in the 8q24 region previously associated with DTC/PTC. However, the association with thyroid cancer was somewhat controversial since the significant association was found in the Polish and UK populations, but no association was found in the Spanish, Italian, and Japanese groups (10). In the Kazakh population under study, we did not observe significant association signal on the whole group analysis, yet a correlation with the higher pT tumor stage was detected. When pT3–T4 tumors were tested, a significant association with PTC risk was confirmed. It is tempting to relegate controversies in the rs6983267 association with thyroid cancer in different populations not only to genetic heterogeneity but also to different distribution of clinicopathological characteristics of tumors in country-specific samples. rs6983267 resides in the intronic region of *POU5F1B*, which encodes a transcriptional activator implicated in multisite cancers (48–51). It is worth noting that this genetic variant is also localized inside the *CCAT2* lncRNA upregulated in colon cancer and implicated in other human malignancies (52–55). The exact roles of either *POU5F1B* or *CCAT2* in PTC remain to be established.

After confirming the associations of rs965513 (*FOXE1*/*PTCSC2*), rs944289 (*PTCSC3*/*NKX2-1*), rs1867277 (*FOXE1*), rs2439302 (*NRG1*), rs10136427 (*BATF*) and, suggestively, of rs966423 (*DIRC3*) with PTC in the whole group or on subgroup analysis for rs7267944 (*DHX35*) and rs6983267 (*POU5F1B/CCAT2*), we combined these genetic variants in a statistical model to evaluate their contribution to PTC risk as the predictive strength of the genetic variants can be improved by combining multiple SNPs in a model (27, 56). The final model, which included four SNPs, rs965513 (*FOXE1*/*PTCSC2*), rs944289 (*NKX2-1*, *PTCSC3*), rs2439302 (*NRG1*) and rs10136427 (*BATF*), was subjected to statistical validation to ensure its reliability. The model had good predictive strength as judged by the ROC analysis (AUC = 0.82). Using two different analogs of the coefficient of determination for logistic regression models, we considered it safe to claim that the four SNPs in the optimal model, adjusted for age and sex effects, could explain about 30–40% of the risk for PTC in the Kazakh population examined in the study with a retrospective case–control design.

Our study had certain advantages such as homogenous ethnicity of the participants, large sample size that provided sufficient statistical power to detect meaningful associations, and thorough selection of the genetic variants. On the other hand, the study was not devoid of limitations. We could not fully rule out sampling bias and acknowledge insufficient age and sex matching of cases and controls, which could affect the accuracy of some of the results obtained in the study. Also, clinicomorphological information was not detailed enough to analyze potentially clinically relevant correlations, and the lack of data on the participants’ lifestyles and environmental exposures impeded the assessment of the impact of these factors on PTC risk.

In summary, our results unambiguously demonstrate the existence of genetic determinants of susceptibility to PTC among the SNPs analyzed in this work in the Kazakh population. We confirm the associations of rs965513 (*FOXE1*/*PTCSC2*), rs944289 (*PTCSC3*/*NKX2-1*), rs1867277 (*FOXE1*), rs2439302 (*NRG1*), and rs10136427 (*BATF*). The association of rs966423 (*DIRC3*) with PTC risk in the Kazakh population is suggestive. The association signals in terms of ORs were generally comparable to those in typical Asian and European populations, and that of rs965513 (*FOXE1*/*PTCSC2*) was the highest so far reported. We also detected the age-related effect of rs1867277 (*FOXE1*) conferring the higher risk for PTC in patients older than 55 years and the association of rs7267944 (*DHX35*) with PTC risk in males and that of rs6983267 (*POU5F1B/CCAT2*) with more advanced tumor pT stage. We estimate the contribution of genetic factors to the susceptibility to PTC in the analyzed series from Kazakhstan to 30–40%, accounting for age and sex. To better understand the impact of different factors affecting PTC risk, further studies would be desirable to increase the number of potential genetic loci and to include the data on individual lifestyle and exposures to environmental agents.

## Data Availability Statement

Dataset used in this study may be made available upon request to Dr. Zhanna Mussazhanova (mussazhanova@nagasaki-u.ac.jp), subject to approval by the Semey Medical University Ethics Committee. Information on all genetic variants analyzed in this study is available in NCBI dbSNP database (https://www.ncbi.nlm.nih.gov/snp/).

## Ethics Statement

The studies involving human participants were reviewed and approved by the Semey Medical University Ethics Committee and Nagasaki University Human Genome Ethics Committee. Written informed consent to participate in this study was provided by the participants’ legal guardian/next of kin.

## Author Contributions

ZM, VS, ME, ZKa, RI, SY, and MN conceived and designed the study. ZM, AK, ME, ZKa, RI, ZY, MM, AM, MS, ZKo, and SB collected and formalized the clinical information. ZM and TR performed the experiments, analyzed and formalized the raw data. VS and HK performed the statistical analyses. TR, SY, KM, and MN contributed comments to the paper. ZM and VS wrote the manuscript. All authors contributed to the article and approved the submitted version.

## Funding

This work was supported in part by the Atomic Bomb Disease Institute of Nagasaki University, The Joint Hiroshima University, Nagasaki University, and Fukushima Medical University Research Base for Radiation Accidents and Medical Science, and by Takeda Science Foundation.

## Conflict of Interest

The authors declare that the research was conducted in the absence of any commercial or financial relationships that could be construed as a potential conflict of interest.
